# Deep phenotyping towards precision psychiatry of first-episode depression — the Brain Drugs-Depression cohort

**DOI:** 10.1186/s12888-023-04618-x

**Published:** 2023-03-09

**Authors:** Kristian Høj Reveles Jensen, Vibeke H. Dam, Melanie Ganz, Patrick MacDonald Fisher, Cheng-Teng Ip, Anjali Sankar, Maja Rou Marstrand-Joergensen, Brice Ozenne, Merete Osler, Brenda W. J. H. Penninx, Lars H. Pinborg, Vibe Gedsø Frokjaer, Gitte Moos Knudsen, Martin Balslev Jørgensen

**Affiliations:** 1 BrainDrugs, Copenhagen University Hospital Rigshospitalet, Copenhagen, Denmark; 2 Neurobiology Research Unit, Copenhagen University Hospital, Rigshospitalet, Copenhagen, Denmark; 3 Department of Clinical Medicine, University of Copenhagen, Copenhagen, Denmark; 4 Psychiatric Centre Copenhagen, Copenhagen, Denmark; 5 Department of Computer Science, University of Copenhagen, Copenhagen, Denmark; 6 Center for Cognitive and Brain Sciences, University of Macau, Taipa, Macau SAR, China; 7 Department of Neurology, Copenhagen University Hospital Rigshospitalet, Copenhagen, Denmark; 8 Department of Public Health, Section of Biostatistics, University of Copenhagen, Copenhagen, Denmark; 9 Center for Clinical Research and Prevention, Bispebjerg & Frederiksberg Hospitals, Copenhagen, Denmark; 10 Department of Public Health, Section of Epidemiology, University of Copenhagen, Copenhagen, Denmark; 11 Department of Psychiatry, Amsterdam UMC, Vrije Universiteit, Amsterdam, the Netherlands

**Keywords:** Major depressive disorder, Biomarker, Synaptic density, PET, MRI, SSRI, EEG, Cognition, Psychotherapy, Precision medicine

## Abstract

**Background:**

Major Depressive Disorder (MDD) is a heterogenous brain disorder, with potentially multiple psychosocial and biological disease mechanisms. This is also a plausible explanation for why patients do not respond equally well to treatment with first- or second-line antidepressants, i.e., one-third to one-half of patients do not remit in response to first- or second-line treatment.

To map MDD heterogeneity and markers of treatment response to enable a precision medicine approach, we will acquire several possible predictive markers across several domains, e.g., psychosocial, biochemical, and neuroimaging.

**Methods:**

All patients are examined before receiving a standardised treatment package for adults aged 18–65 with first-episode depression in six public outpatient clinics in the Capital Region of Denmark. From this population, we will recruit a cohort of 800 patients for whom we will acquire clinical, cognitive, psychometric, and biological data. A subgroup (subcohort I, *n* = 600) will additionally provide neuroimaging data, i.e., Magnetic Resonance Imaging, and Electroencephalogram, and a subgroup of patients from subcohort I unmedicated at inclusion (subcohort II, *n* = 60) will also undergo a brain Positron Emission Tomography with the [^11^C]-UCB-J tracer binding to the presynaptic glycoprotein-SV2A. Subcohort allocation is based on eligibility and willingness to participate. The treatment package typically lasts six months.

Depression severity is assessed with the Quick Inventory of Depressive Symptomatology (QIDS) at baseline, and 6, 12 and 18 months after treatment initiation.

The primary outcome is remission (QIDS ≤ 5) and clinical improvement (≥ 50% reduction in QIDS) after 6 months. Secondary endpoints include remission at 12 and 18 months and %-change in QIDS, 10-item Symptom Checklist, 5-item WHO Well-Being Index, and modified Disability Scale from baseline through follow-up. We also assess psychotherapy and medication side-effects.

We will use machine learning to determine a combination of characteristics that best predict treatment outcomes and statistical models to investigate the association between individual measures and clinical outcomes.

We will assess associations between patient characteristics, treatment choices, and clinical outcomes using path analysis, enabling us to estimate the effect of treatment choices and timing on the clinical outcome.

**Discussion:**

The BrainDrugs-Depression study is a real-world deep-phenotyping clinical cohort study of first-episode MDD patients.

**Trial Registration:**

Registered at clinicaltrials.gov November 15th, 2022 (NCT05616559).

**Supplementary Information:**

The online version contains supplementary material available at 10.1186/s12888-023-04618-x.

## Background

Depression is the leading cause of disability worldwide, with an estimated 322 million people currently suffering from a depressive episode [1]. We know that pharmacotherapy and psychotherapy, individually or combined, can efficaciously treat Major Depressive Disorder (MDD) [2, 3].

Nevertheless, more than half of patients do not respond to the initial medication prescribed [4] and require sequential trials of different treatments that may not alleviate symptoms, resulting in treatment-resistant MDD [5, 6]. After the first depressive episode, approximately half of patients will experience a relapse or re-emergence of depressive symptoms [7]. Current treatment guidelines emphasise depression severity as the primary or exclusive element on which to base treatment choice [8]. Despite numerous studies on definitions, frequency, and determinants of relapse and treatment-resistant MDD [5, 9], we continue to lack clinically relevant markers to guide treatment choice in first-episode MDD.

MDD is highly heterogeneous, with complex disease mechanisms and diverse biological and psychological causes [10], complicating drug discovery and optimal patient care. Deep phenotyping with a subsequent analysis to stratify patients’ response to treatment is an essential tool to resolve this heterogeneity and move away from a ‘one-size-fits-all’ approach [11]. The prospect of this strategy is that patients instead can be stratified based on objective and replicable psychosocial, biochemical and/or neurobiological characteristics. Interventions would ideally be tailored to these individual profiles and thereby maximise clinical response [12]. Furthermore, precision medicine enables the optimisation of resource allocation within patient populations, e.g., by efficient allocation of relapse prevention and interventions to high-risk patient groups.

Previous studies have focused primarily on individual course predictors from just one or two domains, e.g., genetic [13], structural and functional neuroimaging [14–18], electroencephalographic biomarkers [19, 20], blood markers [21], cognitive disturbances [22, 23], and demographic or clinical data [24, 25]. So far, no single marker has proven reliable enough to be implemented in clinical practice [19, 26]. A multimodal approach to depression is essential as the disorder's aetiology is likely multi-causal. That is, prediction models that combine multiple candidate biomarkers might have more predictive utility [24], but must still be generalizable and cross-validated across different cohorts [27]. Other issues include small sample sizes, using data from randomised control trials, lack of clinically and biologically relevant predictive markers, short follow-up time, and poorly validated and biased prediction models [9, 26, 28].

So far, most studies have focused on predictors of response to pharmacological antidepressant treatment without examining the effect of other commonly used treatment modalities such as psychotherapy. Factors such as safety, tolerability, and the effect of the patient's preferences (e.g., dislike of medication or psychotherapy) on overall treatment outcome are also underexplored [26]. Together with substantial methodological variance across studies, these limitations have hampered the ability to draw ecologically relevant and generalizable conclusions from meta-analyses [26, 29, 30].

With this cohort-based study, we will generate a large longitudinal observational multi-modality clinical dataset that allows for a thorough analysis of which phenotypic components enable the best participant stratification for optimal treatment success. We will leverage the Danish healthcare system, which provides standardised treatment packages for all patients. Currently, group cognitive-behavioural therapy (CBT) constitutes the backbone of the treatment package with possibilities for initiation or changes in pharmacotherapy. The treatment package is nationally uniform and designed by Mental Health Services in the Capital Region. The treatment package was introduced in 2013 and revised in 2017 based on clinical and patient experience. However, while the treatment components in the package are research and evidence-based, the treatment effect in real life has not been examined.

The standardised treatment package system enables us to characterise newly diagnosed patients with depression and monitor their clinical response to different treatment modalities, combinations, and paths within the treatment package and deviations from it. Further, the individual Danish person identification number (CPR), combined with several national health and civil registers, will allow us to obtain additional information and follow the patients’ clinical progress longitudinally [31].

### Objectives

The primary objective of this study is to identify single or composite biomarkers that can reliably identify clinical profiles of MDD and predict their treatment outcomes.

**Fig. 1:**
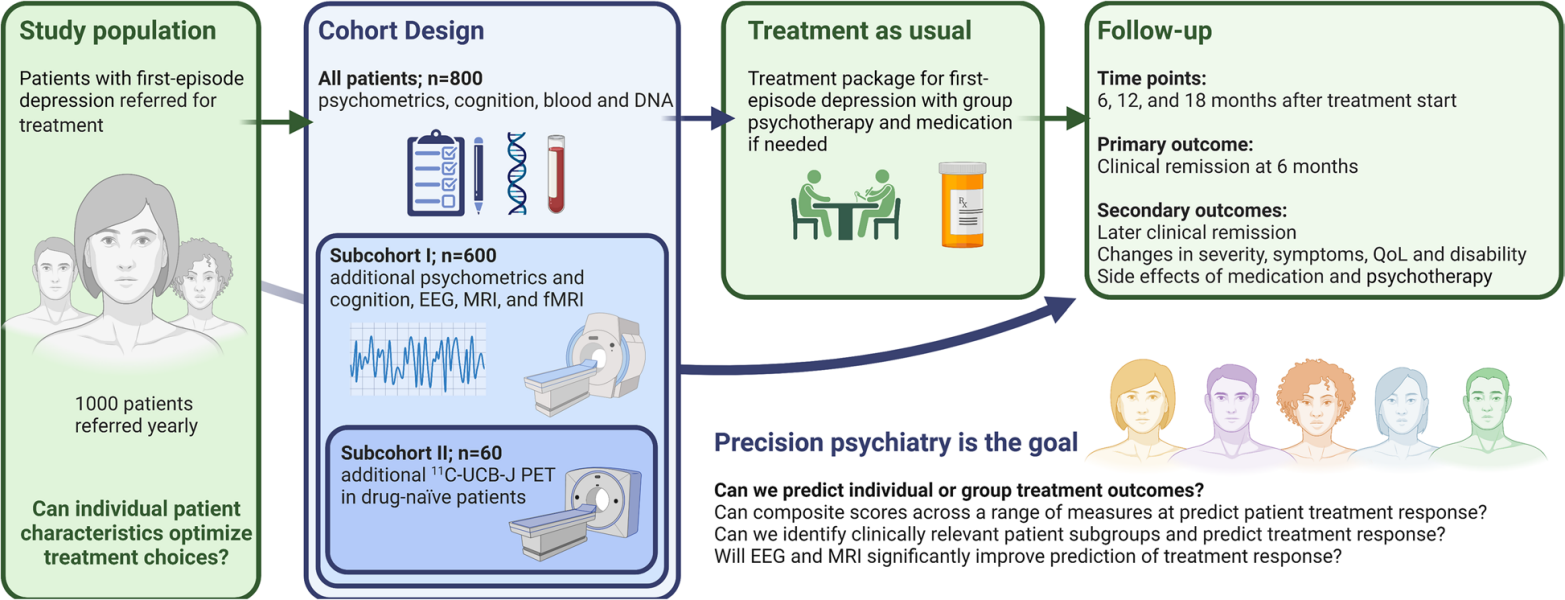
Study design of the BrainDrugs-Depression prospective cohort. *QoL* Quality of life. Figure created by K.H.R.Jensen with BioRender.com

To achieve this aim, we will establish a large, single-site cohort of adult patients diagnosed with first-episode MDD and referred for a treatment package for first-episode depression in secondary care, phenotyping patients before treatment initiation. All patients in the cohort (*n* = 800) will contribute with basic clinical, cognitive, psychometric, and biological data, i.e., genetics and blood biochemistry. A subcohort (subcohort I, *n* = 600) will provide Magnetic Resonance Imaging (MRI) and Electroencephalogram (EEG), and a second subcohort (subcohort II, *n* = 60) of subcohort I with patients unmedicated at inclusion will also undergo a Positron Emission Tomography (PET) brain scan with the presynaptic PET tracer [^11^C]-UCB-J. Clinical depression symptom severity is assessed with the Quick Inventory of Depressive Symptomatology (QIDS) at baseline (T_0_), and 6 (T_1_), 12 (T_2_) and 18 (T_3_) months after treatment initiation (Fig. 1). The treatment package typically lasts six months.

To examine disease trajectories, we will combine collected data with information from Danish national health and social registers, allowing us to further characterise and follow the patients before, during, and after treatment. Together, this might enable us to identify clinically relevant biomarkers and suggest treatment response algorithms, aiding treatment choices and improving patient care (Figure 1).

Furthermore, synaptic loss and deficits in functional connectivity are hypothesized to contribute to depressive symptoms, i.e., cognitive dysfunction, anhedonia, and anxiety and treatment effect. Therefore, we will examine the relationship between presynaptic density and cognitive dysfunction, depressive symptoms, and treatment effect in antidepressant naïve patients (subcohort II) and compare their cerebral presynaptic density to healthy controls (HC).

### Hypotheses

#### The entire cohort

Primary hypotheses:

1.1 Clinical, cognitive, psychometric, genetic, and blood biomarker measures at inclusion can predict clinical remission (defined as QIDS ≤ 5) at the first follow-up.
1.2 Clinical, cognitive, psychometric, genetic, and blood biomarker measures at inclusion can predict clinical improvement (a ≥ 50% reduction in QIDS from pretreatment) at the first follow-up.

Secondary hypotheses:

1.3 Composite scores across a range of clinical, cognitive, psychometric, genetic, and blood biomarker measures at inclusion can cluster patients into MDD subgroups associated with treatment trajectories and outcomes.
1.4 Clinical, cognitive, psychometric, genetic, and blood biomarker measures at inclusion are associated with clinical outcome defined as a change in QIDS.
1.5 Path analysis of baseline patient characteristics and treatment tracks can uncover causal paths for clinical improvements, i.e., estimate the effect of treatment on clinical outcomes.

#### Subcohort I

Primary hypotheses:

2.1 MRI, fMRI, and EEG patterns at inclusion may be associated with depressive phenotypes.
2.2 Adding EEG, MRI, and fMRI measures at inclusion to the classifier model (defined in hypotheses 1.1 and 1.2) may significantly improve the prediction of clinical remission and improvement.

Secondary hypotheses:

2.3 Adding EEG, MRI, and fMRI measures at inclusion to the composite score (defined in hypothesis 1.3) may significantly improve the clustering of patients into MDD subgroups.

#### Subcohort II

Primary hypotheses:

3.1 Cerebral [^11^C]-UCB-J binding is lower in patients with MDD than in healthy controls.
3.2 Domain-specific cognitive function correlates positively with [^11^C]-UCB-J binding in associated cortical and subcortical areas.

Secondary hypotheses:

3.3 Depression severity, anxiety, and anhedonia correlate with [^11^C]-UCB-J binding in associated cortical and subcortical areas.
3.4 Addition of [^11^C]-UCB-J binding, EEG, and MRI measures at inclusion to the composite score (defined in hypotheses 2.1) can significantly improve the prediction of clinical improvement and remission beyond clinical, cognitive, psychometric, fluid biomarker, EEG, and MRI measures in antidepressant naïve patients.

## Methods and design

### Setting

The Capital Region of Denmark has a population of 1.6 million people. Patients in the Capital Region of Denmark are referred by their general practitioner (GP) or other treatment providers to a central diagnostic and referral centre within the mental health services that yearly assesses 20.000 referrals. About 4000 patients are further evaluated in person and diagnosed by the centre.

Five mental health centres in the region provide treatment packages for first-episode depression and will include participants in the study. The Mental Health Centre Amager and the Copenhagen centre consisting of two clinics located in the City of Copenhagen and treat approximately half of all patients, whereas Ballerup and Glostrup treat approximately a third of patients in the surrounding suburb (Sup. Figure 2). The Psychiatric Centre Northern Zealand treats approximately 16% of patients and is located north of Copenhagen, in a region of intermediate urbanisation with individual municipalities classified as rural.

### Study population

We aim to establish a cohort of 800 patients referred to the Danish treatment packages for unipolar first-episode, non-psychotic depression during 2021–2025. We recruit patients from all six clinics in the region. Each clinic receives approximately 100–250 treatment referrals yearly, and approximately 1100 patients are referred yearly. Approximately 80% of referrals are sent directly to the clinics. Patients are recruited during evaluation at the central diagnostic and referral centre or the first consultation in the clinics. Approximately 88% of referrals result in treatment package initiation.

During 2019–2020, 37% of patients were on an antidepressant (usually the selective serotonin reuptake inhibitor (SSRI) Sertraline from their GP) when starting the treatment package, and 54% of patients ended the treatment package on an antidepressant medication. 13% of patients were transferred to a treatment package for a different primary diagnosis group, e.g., generalised, social anxiety, post-traumatic stress disorder, emotionally unstable personality, avoidant personality disorder, eating disorder or obsessive–compulsive disorder. 20% dropped out of treatment. 5% of patients were hospitalised during their treatment package; hospitalization does not preclude the continuation of the treatment package.

The treatment package is a program with manualised psychotherapy in groups of eight patients as the core treatment module together with psychoeducation for the patient and relative (Sup. Table 1). In brief, a treatment package consists of 15–18 h: 2–3 h of initial workup followed by 6 h of individual therapy or 12 sessions of 2 h group therapy (8 patients per group); 1–2 h of engagement and psychoeducation of relatives; 1–5 h of medication clinic; and 2 h of relapse prevention. The program is designed around group-based CBT, but clinics also offer alternatives to CBT, e.g., psychodynamic and schema therapy, and groups for specific demographics, e.g., men or adolescents, and individual therapy. Medication is available as needed.

The research and assessment at baseline for recruited participants is conducted at the Neurobiology Research Unit (NRU) at the Copenhagen University Hospital Rigshospitalet and followed by clinicians from the Mental Health Centre Copenhagen who are not involved in the patient's treatment.

### Inclusion and exclusion criteria for patients

Patients between 18 and 65 years of age referred to a treatment package for single-episode depression will be recruited (Table 1) with minimal exclusion criteria to recruit representative adult outpatients who would typically receive treatment in routine practice (Table 1), of which the majority are women (71%) and aged 18–35 (68%) (S. Figure 1). Patients over 65 (approximately 0.7% of the target population) are excluded because of potential age-related cognitive decline, concomitant medical conditions, or medications that could interact with assessments or treatment (S. Figure 1). Allocation into the subcohorts is based on eligibility, e.g., MRI compatibility, scheduling, and patient willingness to participate.

**Table 1:** Inclusion and exclusion criteria for patients

Patient inclusion criteria:
• Fulfilment of ICD-10 diagnostic criteria for a primary depressive episode (i.e., not secondary to known organic or other psychiatric disorder) • Referral to a treatment package for single-episode depression • Age between 18 and 65 years
**Exclusion criteria:**
• Psychosis or psychotic symptoms • History of severe head trauma involving hospitalization or unconsciousness for more than 5 min • Known, substantial structural brain abnormalities • Insufficient Danish language skills to complete questionnaires and cognitive testing
**Additional exclusion criteria for subcohort I:**
• Severe somatic disease • Contraindications for MRI (e.g., metal implants, claustrophobia, or back problems)
**Additional exclusion criteria for subcohort I:**
• Use of psychotropic drugs • Exposure to radioactivity > 10 mSv within the last year • Pregnancy or breastfeeding

The primary depressive episode, consistent with the International Statistical Classification of Diseases and Related Health Problems version 10 (ICD-10) criteria for MDD without psychotic features (F32.1, F32.2, F32.8 and F32.9), is confirmed by a specialist in psychiatry at the central diagnostic and referral centre.

### Inclusion and exclusion criteria for healthy controls

Data from HCs for comparisons to patients with MDD are available on-site from recent and concurrent projects, stored in the Cimbi database described in [32] including the BrainDrugs-Epilepsy study [33]. Apart from psychiatry-related issues (e.g., no current or history of mental illness or unstable somatic condition), the HC meet the same inclusion and exclusion criteria as required for patients.

### Data collection

**Fig. 2:**
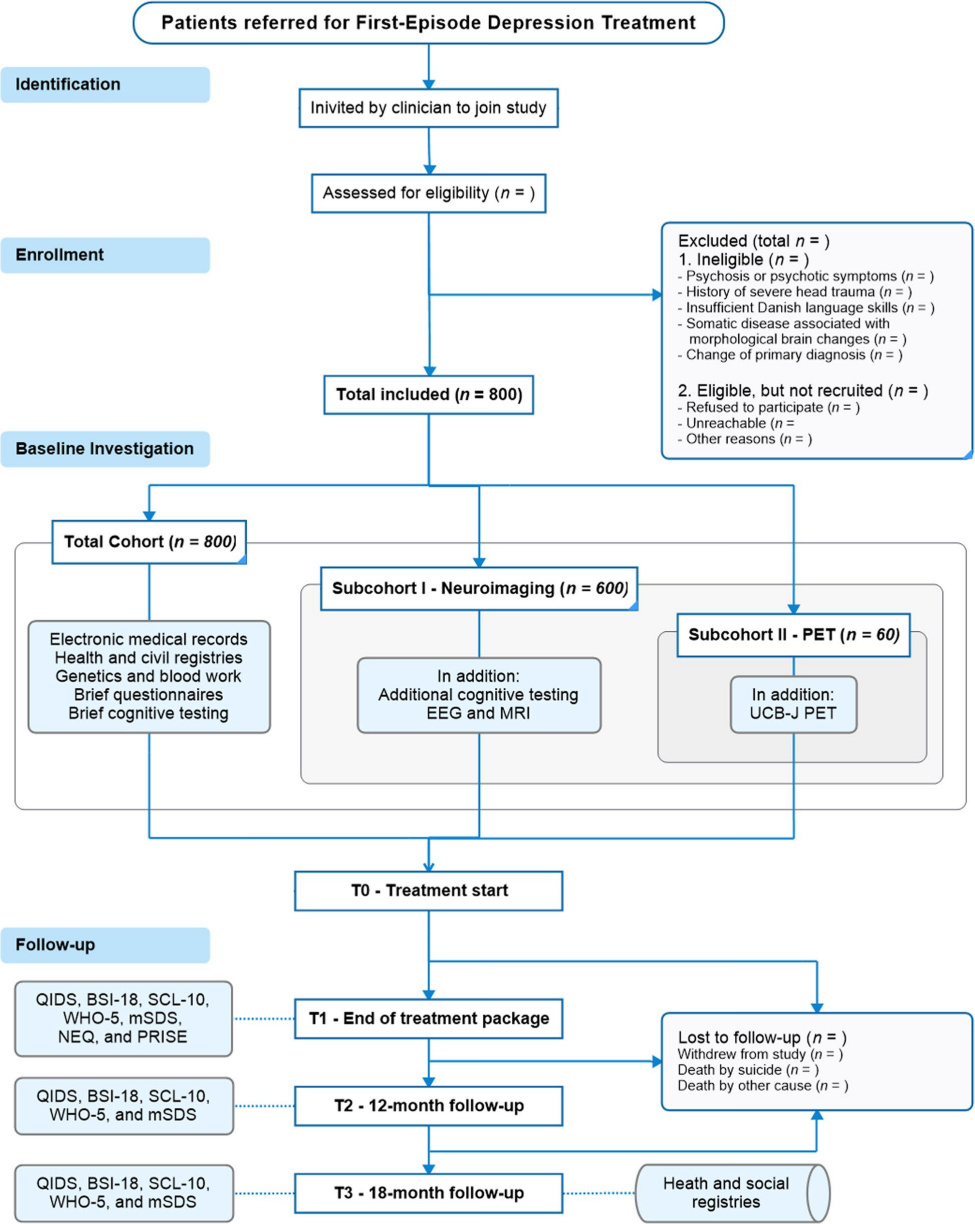
Flow diagram (STROBE) of the BrainDrugs-D cohort study. CVD: (Center for Visitation og Diagnostik) the central referral center within the mental health services in the capital region of Denmark, QIDS-SR: Quick Inventory of Depressive Symptomatology – Self Report, BSI-18: 10 item Brief Symptom Inventory, SCL-10: 10 item Symptom Checklist, mSDS: Modified Sheehan Disability Score, WHO-5: World Health Organisation—Five Well-Being Index (WHO-5), NEQ: Negative Effects Questionnaire, PRISE: Patient-Related Inventory of Side Effects

The patients will undergo a multi-modal investigative program at inclusion and will be followed up after treatment and 12 and 18 months after treatment initiation with questionnaires assessing clinical status (Figs. 1 and 2). Apart from follow-up measures and information extracted from patient files and Danish health and social registries, all data collection will occur before the patient starts treatment.

#### Baseline

##### Questionnaires

Questionnaires will be completed through a secure, web-based survey system hosted by the research centre so that participants can complete questionnaires electronically, either at home or during their visit to the research centre. Online questionnaires are sent via a national secure mail platform used by citizens in regular correspondence with public institutions and the health care system.

Measures include several salient domains in the clinical characterisation of the patient, among others, assessments of demographics (e.g., ethnicity, education, and marital status); medical and psychiatric history; depressive symptoms and impact of depression behaviour and day-to-day life; treatment preferences and expectations, life experiences; and a broad range of state and trait psychometrics. Some questionnaires will only be given to patients in subcohorts I-II (Table 2).

**Table 2:** Questionaries Additional questionnaires for the subcohort I-II only are in bold

Symptom profile and Severity	Cognitive style	Upbringing and life history	Functioning and quality of life
Inventory of Depressive Symptomatology – self-report (IDS-SR) [34]	Mentalisation Questionnaire (MZQ) [35]	Online Stimulant and Family History Assessment Module (OS-FHAM) [11]	Cognitive Complaints in Bipolar Disorder Rating Assessment (COBRA) [36]
Dimension of Anger Reactions (DAR-5) [37]	Ruminative Response Scale (RRS) [38]	Child abuse and trauma scale (CATS) [39]	Modified Sheehan Disability Score (mSDS)
Generalised Anxiety Disorder 7-item (GAD-7) [40]	Perth Alexithymia Questionnaire (PAQ) [41]	**Parental Bonding Instrument (PBI)** [42]	WHO 5 wellbeing index (WHO-5)
Cohen's Perceived Stress Scale (PSS) [43]	**Mindful Attention Awareness Scale (MAAS)** [44]	**Stressful Life Events (SLE)** [45]	Changes in Sexual Functioning Questionnaire (CSFQ) [46]
Brief Symptom Inventory (BSI) [47]	**Short form of Metacognitions Questionnaire (MCQ-30)** [48]		Questions from the Copenhagen Aging and Midlife Biobank (CAMB) [49]
Symptom checklist (SCL-10) [50]	**Coping Self-Efficacy Scale (CSES)** [51]		Revised Sociosexual Orientation Inventory (SOI-R) [52]
Snaith-Hamilton Pleasure Scale (SHAPS) [53]			
**Pittsburgh Sleep Quality Index (PSQI)** [54]			

##### Medical records and registry data

Detailed medical information about previous illness, medication usage, hereditary dispositions, drug, tobacco, and alcohol intake will be acquired for all participants through interviews, self-report questionnaires, electronic medical records (EMR), and registry data.

Data extracted from the EMR will include treatment codes from the MDD treatment package, dates for treatment package start and completion, psychiatric comorbidities; and standard clinical blood work (e.g., HBA1c, TSH, CRP, and cholesterol). In addition, hormonal contraceptive and psychotropic medication prescription and usage (from 1995 onward) will be extracted from the Danish National Prescription Registry [55, 56]. This information includes prescribed medication and dosage and when the patient redeems a prescription. We will retrieve information on lifetime comorbidity from The Danish National Patient Registry (DNPR) [57]. From the Medical Birth Registry, we will obtain data on maternal and maternal perinatal health [58]. We will also collect information on alcohol and drug abuse treatment from the National Registry of Alcohol Treatment and Registry of Drug Abusers Undergoing Treatment. From the social registers in Statistics Denmark, we add data on marital status, occupational history, ethnicity, and educational level [59].

##### Cognitive testing

All patients are assessed with a ~ 1-h neuropsychological test battery, including ‘cold’ (emotion-independent) cognitive tasks indexing reaction time; psychomotor speed; verbal learning and memory; working memory; and executive functions, as well as ‘hot’ (emotion-dependent) cognitive tasks from the Danish version of the EMOTICOM test-battery indexing emotion recognition; emotion detection; and moral emotions in social situations [60].

Patients in subcohorts I-II will complete an additional ~ 1 h of testing with tasks assessing mental flexibility, verbal fluency, and visuospatial learning and memory (see Additional questionnaires for the subcohort I-II only are in bold.

Table 3 for a complete overview of all cognitive tasks). In addition, patients’ subjective experiences of cognitive disturbances will be assessed by the Cognitive Complaints in Bipolar Disorder Rating Assessment (COBRA) questionnaire [36].

**Table 3:** Cognitive testing before treatment

Cognitive Test	Cognitive Domaine
**Whole Cohort**	
Simple Reaction Time task (SRT)	Reaction time
Trail Making Test A & B	Psychomotor speed/executive function
Symbol Digit Modality Task (SDMT)	Psychomotor speed/working memory
Letter-Number Sequence (LNS)	Working memory
D-KEFS Color-Word Interference Test (Stroop)	Executive function
Rey Auditory Verbal Learning Test (RAVLT)	Learning/memory
EMOTICOM Emotional Recognition Task (ERT)	Emotion recognition accuracy
EMOTICOM Emotional Intensity Morphing Task (IMT)	Emotion perceptual detection threshold
EMOITCOM Moral Emotions Task (MET)	Social cognition: guilt and shame
**Additional testing in the subcohorts**	
D-KEFS Verbal Fluency	Executive function
Rey Complex Figure Test (RCFT)	Visuo-spatial learning/memory
Probabilistic Reversal Learning task	Learning within a feedback context
Screen for Cognitive Impairments in Psychiatry—Depression (SCIP-D)	Memory, working memory, vocabulary, psychomotor speed

Patients in subcohorts I-II will complete an additional ~ 1 h of testing with tasks assessing mental flexibility, verbal fluency, and visuospatial learning and memory (see Additional questionnaires for the subcohort I-II only are in bold.

##### Blood biochemistry, genetics, and gene expression

Venous blood samples will be collected for serum, plasma, DNA, and RNA extraction. Identifying biomarkers relevant to the course of depression is an area of research that is evolving rapidly. Thus, based on an ongoing critical literature review, the search for and analysis of specific biomarkers may change during the study period. Currently, the blood biomarkers include inflammation parameters (e.g., high sensitivity CRP) [61, 62] and neurotrophic factors (e.g., BDNF and S100B) [63, 64].

DNA from blood samples will be used for microarray-based genotyping of MDD candidate genes, genes of relevance for MDD (e.g., rs41271330, 5-HTTLPR, COMT, and BDNFval66met), drug metabolism (e.g., CYP2D6, CYP2C19, UGT1A1, ABCB1, ABCC1) and to compute polygenic risk scores in all participants after genome-wide genotyping in the future. DNA will also be used for epigenetic analysis, and circular extrachromosomal DNA, a form of decomposed free DNA [65], will be extracted and characterised. RNA will be extracted for gene transcription profiles using microarray or TAG-based methods (mRNA and microRNA).

DNA from blood samples will be used for microarray-based genotyping of MDD candidate genes, genes of relevance for MDD (e.g., rs41271330, 5-HTTLPR, COMT, and BDNFval66met), drug metabolism (e.g., CYP2D6, CYP2C19, UGT1A1, ABCB1, ABCC1) and to compute polygenic risk scores in all participants after genome-wide genotyping in the future. DNA will also be used for epigenetic analysis, and circular extrachromosomal DNA, a form of decomposed free DNA [65], will be extracted and characterised. RNA will be extracted for gene transcription profiles using microarray or TAG-based methods (mRNA and microRNA).

Gene analyses will be based on a priori models of genetic variations known to modulate pharmacotherapy and psychotherapy responses. The results will be used to calculate a polygenic risk score for diagnosis and treatment response and meta-analyses with established polygenic risk scores for MDD and those currently developed for anxiety and anxiety disorders, including treatment response [66].

##### Electroencephalogram

In subcohort I, we will record resting state EEG and event-related potentials (ERPs) with simultaneous two-lead electrocardiography (ECG) to measure autonomic nervous system activation. EEG will be recorded using a 256-channel HydroCel Sensor Net system (MagstimEGI, USA) at 1000 Hz, where the vertex electrode serves as the reference. Impedances across all electrodes will be kept below 50 kΩ. ECG will be acquired at 1000 Hz using a Physio 16 device (MagstimEGI, USA). EEG/ERP recording: resting EEG (6 min eyes closed and eyes open), two-tone auditory oddball and the LDAEP tasks.

##### MRI

Participants in subcohort I will undergo MRI using a Siemens 3-Tesla Magnetom Prisma scanner. High-resolution structural T1-, T2-, and diffusion-weighted MR images will be acquired as well as ultra-fast functional magnetic resonance encephalography (MREG) asses cardiovascular brain pulsations [67]. Resting-state and task-based blood oxygen level-dependent (BOLD) fMRI scans will be acquired to measure related brain function. To assess distributed and intrinsic brain functional connectivity patterns, we will acquire a resting-state fMRI scan (10 min), during which participants are asked to close their eyes, let their minds wander and not fall asleep. Participants will complete established tasks to assess processes involved in cognition and mood, e.g., the Cyberball task, a ball-tossing game during which the participant interacts with fictitious characters to simulate experiences of social inclusion, exclusion, rejection and ostracism [68]. Trained research personnel will instruct participants on how to perform all tasks.

##### PET imaging

Participants in subcohort II will undergo PET neuroimaging with [^11^C]-UCB-J, which binds to the presynaptic vesicle glycoprotein 2A (SV2A). SV2A is ubiquitously and homogeneously located in synapses across the brain and allows for the determination of SV2A binding and presynaptic density in the brain [69, 70]. However, due to the ubiquitous distribution of SV2A, there is no proper reference region in the brain, and we, therefore, measure the arterial input function. PET scanning is conducted using a High-Resolution Research Tomography (HRRT) PET scanner (CTI/Siemens, Knoxville, TN, USA). First a 6 min transmission scan, then an intravenous bolus of < 400 MBq of [^11^C]-UCB-J administered over 20 s followed by a 90-min dynamic acquisition (256 × 256 × 207 voxels; 1.22 × 1.22 × 1.22 mm).

#### Treatment and life events

As the study is observational, we will not interfere with or delay treatment. EMR and registry data during treatment and until the 18 months from inclusion will be extracted to inform the individual treatment path, i.e., what treatment the individual patient received, e.g., amount of individual psychotherapy sessions and when, timing, type and dose of medication and switches, and participation in group therapy. Using registry data, we can also follow life events, e.g., change of residence, divorce, and employment.

#### Follow-up

Patient-reported outcome measures assessing depression symptom severity and clinical status are sent via the national secure mail platform at three time points: 6, 12 and 18 months after treatment start (Table 4). Complete registry follow-up is done at 18 months as well.

**Table 4:** Follow-up Measurements ^a^Omitted if the patient did not receive antidepressant medication

Measures	6 months	12 months	18 months
Quick Inventory of Depressive Symptomatology (QIDS-SR)	X	X	X
BSI-18, SCL-10, WHO-5 and mSDS	X	X	X
Patient-Reported Inventory of Side-Effects (PRISE)[51]^a^	X	X	X
Negative Effects Questionnaire (NEQ)	X		

##### Primary and secondary outcome measures

The primary clinical outcomes are categorical: *improvement* defined as ≥ 50% reduction in QIDS and *remission* after the treatment package defined as a QIDS ≤ 5. The secondary outcome is *change* in depression severity as measured by QIDS.

##### Tertiary outcome measures

The ten-item depression and anxiety symptom checklist (SCL-10) [50, 71], well-being measured by WHO-5 [50, 72], and disability measured by a modified Sheehan Disability Scale (mSDS) are the established treatment effect parameters by the Mental Health Services of the Capital Region of Denmark.

Tertiary endpoints are three measurements of psychosocial remission defined as a WHO-5 score of > 49, an SCL-10 score of < 26 and an mSDS score of < 10. Additional tertiary clinical endpoints are changes in wellness (WHO-5), disability (mSDS), and symptomatology on the Brief Symptom Inventory 18 (BSI-18) and the SCL-10.

Two questionnaires assessing the negative effects of psychological and antidepressant treatment will be sent at the first follow-up, at the end of the treatment package. We use the Patient-Reported Inventory of Side-Effects (PRISE), originally developed and validated in Danish and also used in the STAR*D trial [71, 73]. The questionnaire is omitted if the patient has not been or is not on antidepressant medication. We use the short 20-item form of the Negative Effects Questionnaire (NEQ) to assess adverse and unwanted events in psychological treatment, i.e., new symptoms, dependency, stigma, hopelessness, and the experienced quality of treatment [74, 75]. Both baseline characteristics and treatment experiences, e.g., negative effects on the NEQ, will be used to investigate reasons for CBT and treatment package drop-out.

##### Registry follow-up

After the last 18-month follow-up, the study dataset will be sent to Statistics Denmark with a list of all invited participants to allow a non-participant analysis and long-term follow-up. The study data will be linked with data from the Danish Civil Registration System [76] e.g., the DNPR [57], the Danish National Prescription Registry [55, 56], and other registries indexing, e.g., hospital admittance, diagnosis- and treatment codes, prescription medications, employment status, living situation (e.g., partner information), and income. We will also examine diagnostic stability [77], e.g., change of primary diagnosis from first episode depression to an anxiety or personality disorder or later recurrent depressive episode or conversion to bipolar affective disorder.

### Statistical analyses

We plan to investigate the outlined hypotheses using both data- and hypothesis-driven approaches. We will publish more specific analysis plans on the web (e.g., PROSPERO) before starting detailed analyses.

#### Data-driven analyses

We will use machine learning algorithms to determine a combination of baseline characteristics that best predict treatment outcomes. In contrast to hypothesis-driven analyses, data-driven machine learning frameworks enable us to identify novel associations between patient characteristics and treatment response and interactive effects of complementary patient characteristic information in prediction. Recent interest in machine learning approaches to prediction is to develop *classifiers* that combine a range of patient-level data to provide a patient-level prediction of treatment response. The most common classifiers are based on patient characteristics or biomarkers assessed before treatment initiation [78, 79]. This information may be used in clinical settings to select a specific treatment if it is predicted to have a higher chance of success or to suggest that a patient may generally be treatment-resistant, which could justify earlier use of second-line therapies [26].

In the present study, we will first use machine learning to train classifiers based on broad non-imaging data collected from all patients in the cohort. Neuroimaging data from EEG, MRI, and PET in subcohorts I and II will subsequently be included, enabling us to make meaningful statements about the marginal improvement in model performance with or without specific neuroimaging measures. This is critical for optimising patient care with costs associated with data acquisition. Following the recommendations for best practice [80, 81], we will employ randomized k-fold nested cross-validation, i.e., splitting the collected data into training and testing datasets that reported model performance measures are not upwardly biased due to data leakage.

We will use latent class analysis to identify different MDD subgroups that share characteristics measured at baseline (hypotheses 1.2 and 2.3) [25, 82, 83]. To investigate if adding neuroimaging data improves clustering (hypothesis 2.3), we will compare the heterogeneity of change on clinical outcome, i.e., mean and variance.

#### Hypothesis-driven analyses

To answer the hypothesis-driven research questions, i.e., hypotheses 3.1 – 3.4, we will use appropriate parametric models, including multiple linear regression or linear latent variable models to investigate group differences in [^11^C]-UCB-J binding between patients and HCs and multiple linear regression to determine associations between cognitive scores and [^11^C]-UCB-J binding in patients. We will adjust for age and sex, as our current data on HCs with [^11^C]-UCB-J are of equal sex distribution with a mean age of approximately 30 years old, which is not expected for the patient sample expecting to be predominately women and below 25 years old (Supplementary Fig. 1). Furthermore, dependent on additional funding, we will include more HCs and attempt to match their age distribution with the patients better.

We will also use statistical models such as logistic regression to investigate associations between individual measures and dichotomous clinical outcomes (e.g., the association between early childhood trauma and treatment response) when testing secondary hypotheses. Lastly, we will assess the associations between baseline patient characteristics, treatment events, and clinical outcomes using path analysis, enabling us to estimate the effect of treatments over time on the clinical outcome.

### Power calculation

#### Data-driven analyses

To answer primary hypotheses 1.1 – 3.1, we will use a machine learning approach as outlined above. Statistical power calculations are not well adapted to data-driven machine learning model frameworks because any such calculation depends on broad assumptions about model structure and feature space. However, the number of patients in the cohort (*N* = 800) represents one of the largest cohorts to date, looking to determine prediction classifiers in MDD [84].

To limit the strain on patients as well as costs, we collect neuroimaging data in a subset of patients (*N* = 600) and not in the entire cohort. By contrast, questionnaire and EMR data collection is relatively cheap, fast, and non-invasive. Therefore, this data's potential predictive value need not be very high to be clinically relevant. Acquisition of neuroimaging data, i.e., MR and PET, is costly and time-consuming and must exhibit higher predictive value to be relevant as a clinical tool. Thus, fewer patients are needed in Cohorts II-III to determine the relevance of neuroimaging biomarkers in MDD, as the power of these biomarkers would have to be large enough to be detectable even in smaller patient samples.

#### Hypothesis-driven analyses

To answer hypothesis 3.1, we will use [^11^C]-UCB-J PET data from healthy controls (*N* = 40) currently available from the Cimbi database with [^11^C]-UCB-J PET data collected from patients in the PET subcohort II (expected *n* = 60). These sample sizes will provide us with a statistical power of 0.99 to detect group differences with a Cohen's *d* effect size of 0.95 as reported in previous study [85]. With the samples size and a statistical power of 0.80 we can defect a group difference with a Cohen’s *d* 0.58 or higher using a significance threshold of *p* ≤ 0.05 in a two-sample *t*-test. The previous study by Holmes et al. (2019) reported group differences in [^11^C]-UCB-J binding between healthy controls and a small cohort with mixed psychiatric diagnoses, including MDD. Based on their findings in frontal cortex binding (which were similar to other brain regions), our study is statistically powered to detect group differences in binding of ~ 6.8%; notably, Holmes et al. found a group difference of 12.5% in this region, so our study should be adequately powered.

To answer hypothesis 3.2, we will have a statistical power of 0.8 to detect a significant association between [^11^C]-UCB-J binding and cognitive scores in the PET subcohort II equivalent to a correlation coefficient of *r* ≥ 0.35 at a statistical significance threshold of *p* ≤ 0.05.

### Ethics and data availability

The study is conducted according to the principles of the seventh revision of the Declaration of Helsinki (2013) and was reviewed and approved by the Committees on Health Research Ethics in the Capital Region of Denmark (reference number: H-20083013). Before undertaking any study-related procedures, each participant receives verbal and written explanations of study aims, methods, potential hazards, and benefits from investigators and provides written informed consent. All participating patients are asked to consent to disclose relevant information from their EMR to extract health-related information relevant to the study.

Data management and monitoring during the study adhere to the rules protecting personal data. Paper-based material (e.g., cognitive test results) will be stored in a secured archive. Identifiable electronic data files will be stored in password-secured files behind a firewall per regulations.

Biological material will be coded with a unique identification number. Access to de-identification keys is restricted to authorised personnel only and stored in a temporary biobank located in secured areas in the laboratory facility. The biomaterial will later be analysed in batches to reduce noise, and potential extra material after the end of the study will be transferred to the CIMBI biobank [32]. All biological material will ultimately be anonymised after 15 years after the end of the study.

The study results will be presented following relevant reporting guidelines, i.e., STROBE and TRIPOD [86, 87]. After publication of results from our primary hypothesis, the data can upon request be made available to other scientists or consortia, through the Cimbi database (or similar platform).

## Discussion

MDD is a brain disorder with etiological heterogeneity in the interplay between biological, social, psychological, and behavioural factors and pathophysiology with several neurobiological mechanisms. In the BrainDrugs-Depression study, we employ a broad, multi-modal biopsychosocial characterisation of each patient. This, combined with a large sample size, follow-up over 18 months, and further complete follow-up in the social- and health registries, offers a unique opportunity to uncover potential single or combined predictors of treatment outcome, identify MDD subtypes, advance the understanding of MDD aetiology, and map neurobiological predictors of treatment response; ultimately paving the way for a precision medicine approach for optimised MDD treatment.

Further, the ability to track and follow patients in the Danish health care system and registers provides a unique opportunity to obtain large amounts of data collected independent of the patient’s mood and enables us to perform sensitivity analyses and account for selective participation and attrition. The study also makes it possible to track the long-term consequences of various degrees of treatment response and point to areas where the current treatment could be improved to obtain better prognostic outcomes.

Cognitive dysfunction is a core dimension in MDD, both in first- and multiple-episode patients [88] and mediates a significant degree of psychosocial impairment and reduction in workplace productivity [89]. The presence of cognitive dysfunction may also significantly impact medication [90] and psychotherapy [91] and may even persist even past remission of the depressive episode [92, 93]. Recently, researchers have begun to explore the potential of cognitive markers to inform clinical decision-making in the treatment of depression [94] and as a specific treatment target [95]. We include an extensive neurocognitive battery at inclusion and hope to extend the follow-up assessments to include a brief internet-based self-administered cognitive assessment [96] to follow long-term cognitive function.

This study is embedded in a more extensive network of closely related studies in the BrainDrugs Research Alliance (braindrugs.nru.dk), which will significantly increase its scientific scope and value. Specifically, a concurrent prospective cohort study, the BrainDrugs-Epilepsy study at our research unit, studies newly diagnosed patients with epilepsy using a similar multi-modal precision medicine approach [33]. Depression and other psychiatric disorders (e.g., anxiety) are frequent in patients with epilepsy, and a bidirectional relationship has been proposed [97–100]. Shared measures enable further exploration of the relationship between depression and epilepsy.

During the last decades, new developments in the pharmacological treatment of depression have been modest. Emphasis has been paid to the effect of other treatment modalities, such as psychotherapy, lifestyle modification and a combination of treatments. However, we do not know which specific groups of patients benefit from different treatment modalities. In the present project, we develop tools for prediction and uncover causal paths for treatment response in a real-world setting. Thus, the project goes beyond a traditional evaluation of existing health services with the potential to develop a more targeted treatment to be implemented and tested in the clinical setting. Furthermore, to maximise the utility of the participants’ contribution, the project in also intended for cross-validating models from other research groups, data sharing and multi-centre collaborations. The datasets generated by this study will be available in the Cimbi database, which researchers can request access to [32].

## Supplementary Information

**Additional file 1: Supplementary Figure 1. **The age and sex distribution of patients enteringthe treatment package during 2019-2021 before inclusion start.

**Additional file 2: Supplementary Figure 2. **The annual distribution of treatment initiation at the Mental Healthcare Centres in the Capital Region of Denmark. *The Mental Health Centre Copenhagen comprises two clinics, i.e., in Frederiksberg and Nørrebro. B) The Mental Health Centres admission area and geographical locations (image made by K. R. Jensen).

**Additional file 3.**

## Data Availability

The datasets generated by this study will be available in the Cimbi database. All researchers can request access to data from the Cimbi database (www.cimbi.dk/db).

## Abbreviations

5-HTTLPR: Serotonin-transporter-linked promoter region
ABCB1: ATP Binding Cassette Subfamily B Member 1
ABCC1: ATP Binding Cassette Subfamily C Member 1
BDNF: Brain Derived Neurotrophic Factor
BOLD: Based blood oxygen level-dependent
BSI: Brief Symptom Inventory
BSI-18: 18-Item Brief Symptom Inventory
CAMB: Copenhagen Aging and Midlife Biobank
CATS: Child abuse and trauma scale
CBT: Cognitive behavioral therapy
CIMBI: Center for Integrated Molecular Brain Imaging
COBRA: Cognitive Complaints in Bipolar Disorder Rating Assessment
COMT: Catechol-O-methyltransferase
CPR: Centrale Personregister
CSES: Coping Self-Efficacy Scale
CSFQ: Changes in Sexual Functioning Questionnaire
CVD: Center for Visitation og Diagnostik
CYP2C19: Cytochrome P450 Family 2 Subfamily C Member 19
CYP2D6: Cytochrome P450 Family 2 Subfamily D Member 6
DAR-5: Dimension of Anger Reactions
D-KEFS: Delis-Kaplan Executive Function System
DNA: Deoxyribonucleic acid
DNPR: Danish National Patient Registry
EEG: Electroencephalogr
EMR: Electronic medical records
ERP: Event-related potentials
ERT: Emotional Recognition Task
GAD: Generalised Anxiety Disorder 7-item
GP: General practitioner
HBA1c: Hemoglobin A1C
HC: Healthy control
HRRT: High resolution research tomograph
ICD: International Classification of Diseases
IDS: Inventory of depressive symptomatology
IMT: Emotional Intensity Morphing Task
LDAEP: Loudness dependence of auditory evoked potentials
LNS: Letter-Number Sequence
MAAS: Mindful Attention Awareness Scale
MCQ-30: Short form of Metacognitions Questionnaire
MDD: Major Depressive Disorder
MET: Moral Emotions Task
MREG: Ultra-fast functional magnetic resonance encephalography
MRI: Magnetic Resonance Imaging
mSDS: Modified Sheehan Disability Scale
MZQ: Mentalization Questionnaire
NEQ: Negative Effects Questionnaire
NRU: Neurobiology Research Unit
OS-FHAM: Online Stimulant and Family History Assessment Module
PAQ: Perth Alexithymia Questionnaire
PBI: Parental Bonding Instrument
PET: Positron emission tomography
PRISE: Patient Reported Inventory of Side-Effects
PSQI: Pittsburgh Sleep Quality Index
PSS: Cohen's Perceived Stress Scale
QIDS: Quick Inventory of Depressive Symptomatology
RAVLT: Rey Auditory Verbal Learning Test
RCFT: Rey Complex Figure Test
RRS: Ruminative Reponse Scale
S100B: S100 calcium-binding protein B
SCIP-D: Screen for Cognitive Impairments in Psychiatry—Depression
SCL-10: 10-Item depression and anxiety Symptom Checklist
SDMT: Symbol Digit Modality Task
SHAPS: Snaith-Hamilton Pleasure Scale
SLE: Stressful life events
SOI-R: Revised Sociosexual Orientation Inventory
SRT: Simple Reaction Tim
SSRI: Selective serotonin reuptake inhibitor
STROBE: Strengthening the reporting of observational studies in epidemiology
SV2A: Synaptic vesicle glycoprotein 2A
TRIPOD: Transparent Reporting of a multivariable prediction model for Individual Prognosis Or Diagnosis
TSH: Thyroid-stimulating hormone
UGT1A1: UDP glucuronosyltransferase 1 family, polypeptide A1
WHO: World Health Organization
WHO-5: WHO 5 wellbeing index
