# Supplementary figures and images for: Deep phenotyping towards precision psychiatry of first-episode depression — the Brain Drugs-Depression cohort
Source: BMC Psychiatry. 2023 Mar 9;23:151. doi: 10.1186/s12888-023-04618-x (PMC9999625; doi:10.1186/s12888-023-04618-x)

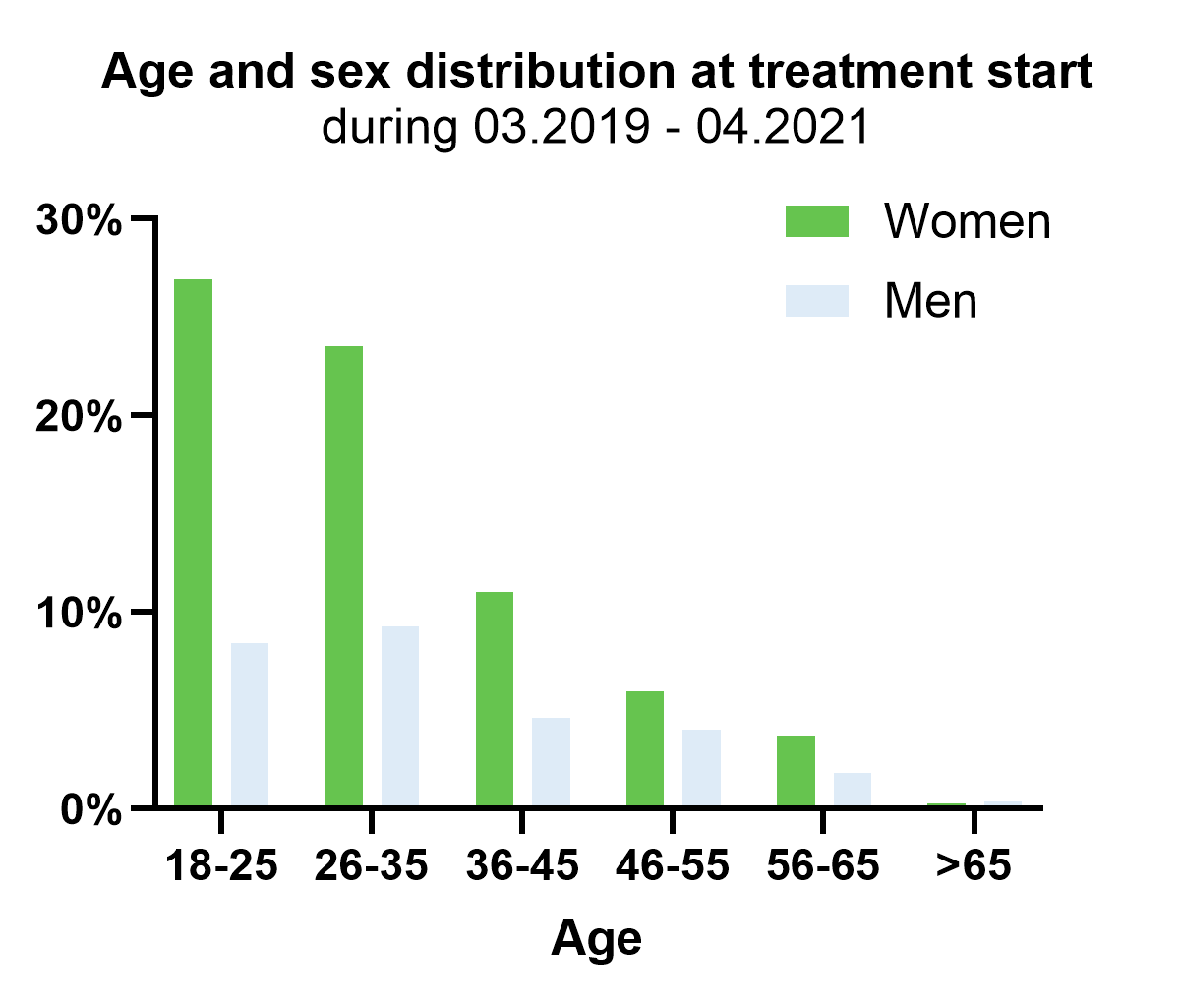

Supplement: Supplementary file 1 — Additional file 1: Supplementary Figure 1. The age and sex distribution of patients enteringthe treatment package during 2019-2021 before inclusion start. [file 12888_2023_4618_MOESM1_ESM.png]

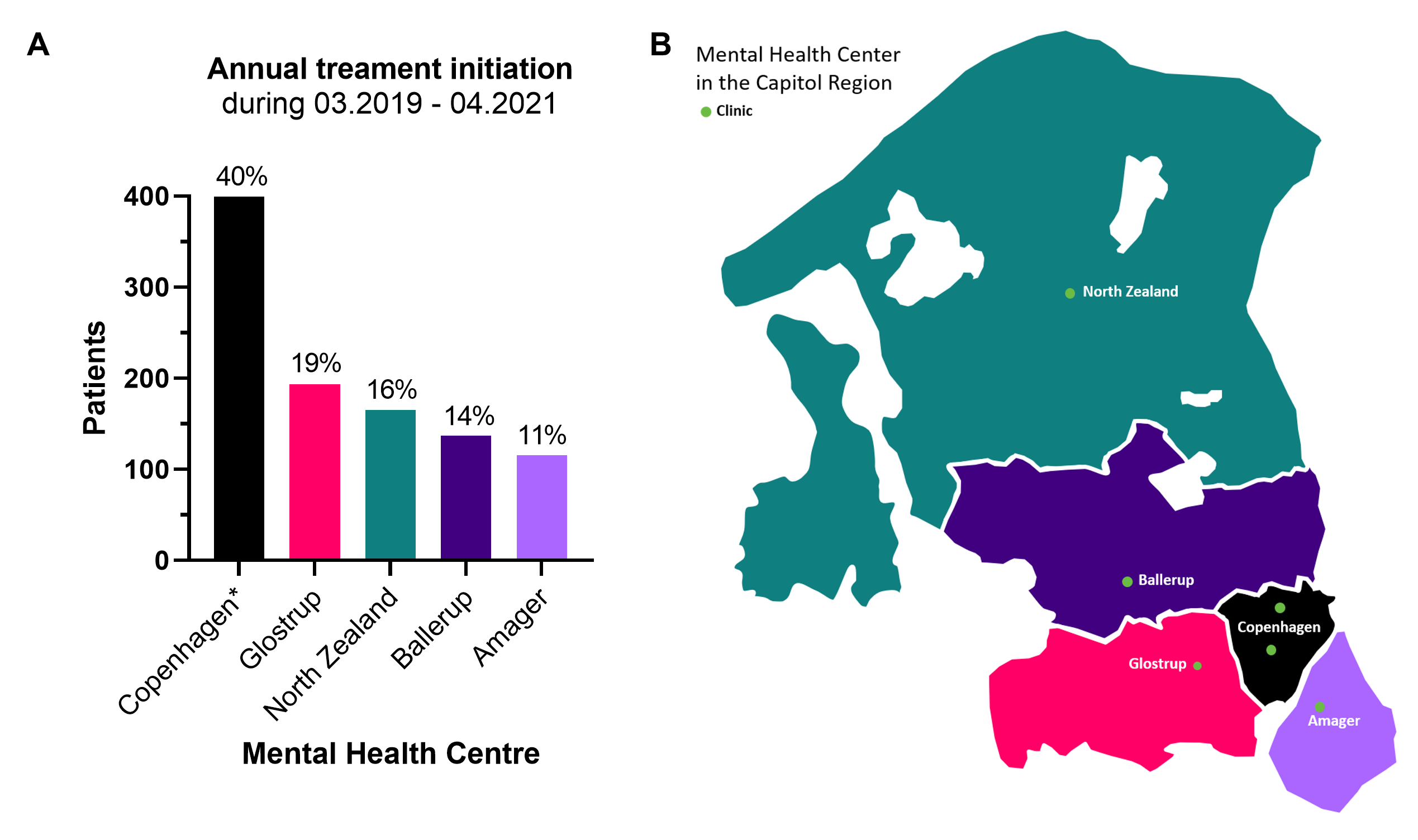

Supplement: Supplementary file 2 — Additional file 2: Supplementary Figure 2. The annual distribution of treatment initiation at the Mental Healthcare Centres in the Capital Region of Denmark. *The Mental Health Centre Copenhagen comprises two clinics, i.e., in Frederiksberg and Nørrebro. B) The Mental Health Centres admission area and geographical locations (image made by K. R. Jensen). [file 12888_2023_4618_MOESM2_ESM.png]
