# Supplementary material for: Deep phenotyping towards precision psychiatry of first-episode depression — the Brain Drugs-Depression cohort
Source: BMC Psychiatry. 2023 Mar 9;23:151. doi: 10.1186/s12888-023-04618-x (PMC9999625; doi:10.1186/s12888-023-04618-x)
Supplement: Supplementary file 3 — Additional file 3. [file 12888_2023_4618_MOESM3_ESM.docx]

**Supplementary Table 1**

| **Treatment** | | **Cumulative Time** | **Content** |
| --- | --- | --- | --- |
| **Examination and Monitoring** | Initial examination | 2 hours | The patient is seen and assessed by a specialist  in psychiatry  Investigation of psychopathology with diagnostic interview |
|  | Psychometric  monitoring | 1 hour | Hamilton Depression Scale rating |
|  | Continuous examination |  | Examination of cognitive function in remitted phase and in case of suspected cognitive difficulties  Investigation of social support needs  Somatic and neurological examination |
| **Non-pharmacological Treatment**  (in groups and individually as needed) | Individual | 6 hours  *as required* | Psychotherapy  - Cognitive behavioural therapy or other similar short-term therapy  - Psychoeducation |
|  | In group (e.g., 8 patients) | 6 hours  *as required*  *(12 sessions  of two hours)* | Psychotherapy  - Cognitive behavioural therapy or other similar short-term therapy  Behavioural training  Psychoeducation |
|  | Relatives | 2 hours | Relative involvement  Psychoeducation |
| **Pharmacological Treatment** | | 5 hours | Treatment with antidepressants  Additional treatment  Restrictions on the use of benzodiazepines  Monitoring of side effects and metabolic disorders  Systematic adverse reaction detection |
| **Network** | | 2 hours | Network meetings with coordination of efforts  - Contact with partners, etc.  Follow-up on treatment and relapse prophylaxis |
